# Stannous Fluoride Preventive Effect on Enamel Erosion: An In Vitro Study

**DOI:** 10.3390/jcm9092755

**Published:** 2020-08-26

**Authors:** Alessandra Lucchese, Angelica Bertacci, Antonino Lo Giudice, Elisabetta Polizzi, Enrico Gherlone, Maurizio Manuelli, Stefano Chersoni, Daniele Moro, Giovanni Valdrè

**Affiliations:** 1 Department of Dentistry, Dental School, IRCCS San Raffaele Hospital Vita Salute San Raffaele University, 20123 Milan, Italy; luigitorsello@libero.it (E.G.); maurizio.manuelli@libero.it (M.M.); 2 Unit of Dentistry, Research Center for Oral Pathology and Implantology, IRCCS San Raffaele Scientific Institute, 20123 Milan, Italy; 3 Department of Biomedical and Neuromotor Sciences (DiBiNeM), School of Dentistry, University of Bologna, 40126 Bologna, Italy; angelica.belocci@unibo.it (A.B.); stefano.chersoni@unibo.it (S.C.); 4 Department of General Surgery and Surgical-Medical Specialties, School of Dentistry, University of Catania, Policlinico Universitario “Vittorio Emanuele—G. Rodolico”, Via S. Sofia 78, 95123 Catania, Italy; nino.logiudice@gmail.com; 5 Center for Oral Hygiene and Prevention, Dental School, Vita-Salute San Raffaele University and IRCCS San Raffaele, 20123 Milan, Italy; marcolinamarta@libero.it; 6 Department of Biological, Geological, and Environmental Sciences, University of Bologna, 40126 Bologna, Italy; daniele.moro@unibo.it (D.M.); giovanni.valdre@unibo.it (G.V.)

**Keywords:** oral health, quality of life, enamel erosion, calcium, imaging

## Abstract

The aim of this in vitro study was to evaluate the effects of a single dose application of two daily toothpastes on enamel exposed to acid attack. The research was conducted on human molars enamel fragments (*n* = 72). The two different toothpastes active ingredients were sodium fluoride (NaF) and stannous fluoride (SnF_2_). They were compared in protecting the surface of the enamel exposed to three acids: citric acid, lactic acid and hydrochloric acid. A spectrophotometer was used to measure the calcium ions and phosphate released in the solutions by the enamel specimens. Afterward, ionic concentrations were analyzed through the t-Student test, in order to estimate the significance level (*p* < 0.05) of the solubility differences obtained between the treatment and control groups. Finally, sample surfaces were analyzed with scanning electron microscopy and X-ray energy dispersive spectroscopy (SEM/EDX). The two analyzed toothpastes did not reveal any statistically significant variation in the release of calcium and phosphate (*p* > 0.05). Nevertheless, acid-resistant deposits were detected in samples treated with stannous fluoride and exposed to lactic acid, though the presence of tin ion deposits on samples treated with stannous fluoride was not shown. A single dose of a fluoride-based toothpaste before different acids attack, in simulated oral cavity conditions, did not show a significant preventive effect.

## 1. Introduction

Dental erosion is defined as the result of a chronic, localized and irreversible pathological loss of hard tooth tissues and is caused by chemical-like processes, without the involvement of microorganisms [1]. The prevalence and incidence of dental erosion have increased steadily in recent years, involving about a third of the population of the western world [2], especially in younger age groups and in the male population [3]. Soft drinks widely consumed among children and adolescents in Western and developing countries are able to cause demineralization of large enamel areas, as demonstrated using scanning electron microscopy (SEM) [4,5,6,7]. Clearly, the erosion problem does not affect prosthetic teeth and dental implants [8,9] but could affect brackets retention [10,11] and increase the incidence of white spot lesions in orthodontic patients [12].

The buffering effect of saliva cannot neutralize acidic compounds, and preventive strategies for dental erosion is necessary. This will have a great effect on orthodontics with a fixed appliance, improving the shear bond strength between the tooth and the brackets [13,14,15,16].

Changing the patient’s diet and applying a fluoride toothpaste are two solutions [17]. Although the sodium fluoride (NaF) is the main ingredient of caries preventing toothpaste, it has a limited effect in preventing erosion [18]. The true inhibitor of dental erosion is the stannous ion [2,19,20,21].

Low pH in the oral cavity involved enamel loss of calcium and phoshate ions [22]; spectrophotometric analysis of the release of calcium and phosphate in solution has been used in the literature to evaluate the outcomes of acid attack of the hard tissues of the tooth, and represents a reliable and reproducible analysis method [23].

This study aims to quantitatively evaluate the effect of a stannous fluoride toothpaste in comparison with a traditional sodium fluoride-based toothpaste on the enamel exposed to acid attack. The null hypothesis is that a single application of sodium fluoride and stannous fluoride does not modify the release of calcium and phosphate in solution from enamel exposed to acid attack with citric, hydrochloric and lactic acid.

## 2. Experimental Section

Enamel fragments (*n* = 72), with a weight of approximately 0.3 gr, were used for this study. They were obtained with a diamond bur (Isomet, Buehler Ltd., Lake Bluff, IL, USA) from 36 caries-free human molars (patients age 18–25 years), extracted for orthodontic reasons. The study was conducted in accordance with the Declaration of Helsinki, and the protocol was approved by the Ethics Committee of IRCCS San Raffaele Scientific Institute, Milan, Italy (107/1NT/2017).

### 2.1. Samples Organisation

The pairs of fragments from each tooth were immersed into a storage solution of double distilled water (Carlo Erba, Cornaredo, Italy) with a modified pH value of 7.4 at 4 °C, prior to their use.

The specimens were organized in two groups of 36 pieces (18 pairs), and pair samples of each group were split into a case group (*n* = 18) and a control group (*n* = 18), respectively, in order to compare enamel from the same tooth.

Each case group (*n* = 18) provided for teeth brushing for 2 min with an electric brush with pressure control (Oral-B Triumph, Procter & Gamble, Cincinnati, OH, USA). The toothpaste dose was controlled (1 g). Each control group (*n* = 18) did not follow any kind of treatment.

The two case groups tested two different commercial toothpastes, as follows:

- Group A (*n* = 18): AZ ProExpert^®^, Procter & Gamble, Cincinnati, Ohio with 1100 ppm SnF_2_ and 350 ppm NaF;
- Group B (*n* = 18): Colgate Total Original^®^, Colgate-Palmolive, New York, NY, USA with 1450 ppm NaF.

After brushing, the cases specimens were rinsed with deionized water for 10 s and stored in artificial saliva (1.5 mmol/L CaCl_2_, 50 mmol/L KCl, 0.9 mmol/L KH_2_PO_4_ Tris, pH 7.4) at 37 °C for 2 h.

After storage, specimens of each main group (*n* = 36 cases and *n* = 36 respective controls from the same tooth) were assigned to three subgroups (*n* = 12), respectively, and immersed for 5 min in an acid solution. The three solutions contained, respectively, citric acid (pH 1.78), hydrochloric acid (pH 2.15) and lactic acid (pH 2.3).

These three acids were tested because they are recognized as the most frequent causes of erosion and demineralization of the enamel.

### 2.2. Measurements

The quantitative evaluation of enamel demineralization is carried out through the spectrophotometric measurement of calcium and phosphate ions released in the solution. Measurements were carried out by using a spectrophotometer (Perkin Elmer Lambda 25) with quartz cuvette and with Diagnostic Kit (Hagen Diagnostika, Hagen, Germany) for calcium ions (cod. 001–0037) and for phosphate (cod. 001–0017). In order to normalize the data, the values of the obtained ionic concentrations were standardized to the weight of each single fragment (0.30 g ± 0.02).

### 2.3. Statistical Analysis

To test the significance level of the solubility’s differences between the treatment and control groups on the outcome variables, the t-Student test was performed. In this way, the calcium (Ca^2+^) and phosphate (PO_4_^3−^) ions release was analyzed from the dissolution tests. The *p*-values reported as statistically significant was <0.05. The α threshold was set at 0.05.

### 2.4. SEM/EDX Analysis

After the treatments, all the samples were subjected to graphite metallization for Scanning Electron Microscopy (SEM) with Energy Dispersive X-ray Analysis (EDX). For this purpose, the correct analytical strategy for the particular sample must be taken into consideration [24]. The SEM/EDX analyses were conducted by using both a SEM Jeol JSM 5400 equipped with IMAGESLAVE^®^ and an EDS IXRF system (IXRF, Inc., Austin, TX, USA), and a SEM Philips XL 20 with EDS EDAX-DX-4. This analysis was performed to examine the enamel surface morphology, the presence of deposits on the enamel surface and their chemical composition.

## 3. Results

### 3.1. Enamel Dissolution Analysis

All the results are reported in Table 1, Table 2 and Table 3.

No statistically significant differences (*p* > 0.05) were found in calcium and phosphate release difference (∆%) between the two examined toothpaste and between the cases and the controls groups.

No statistically significant differences were found between the means values of calcium and phosphate release between the two examined toothpaste and between the cases and the controls groups (*p* > 0.05) for citric and lactic acid treated samples. Statistically significant differences were found only in the group treated with NaF, between the case and the control groups (*p* < 0.05); the untreated samples (controls) showed a low release of phosphate and calcium after immersion in hydrochloric acid.

### 3.2. SEM/EDX Analysis

**Figure 1:**
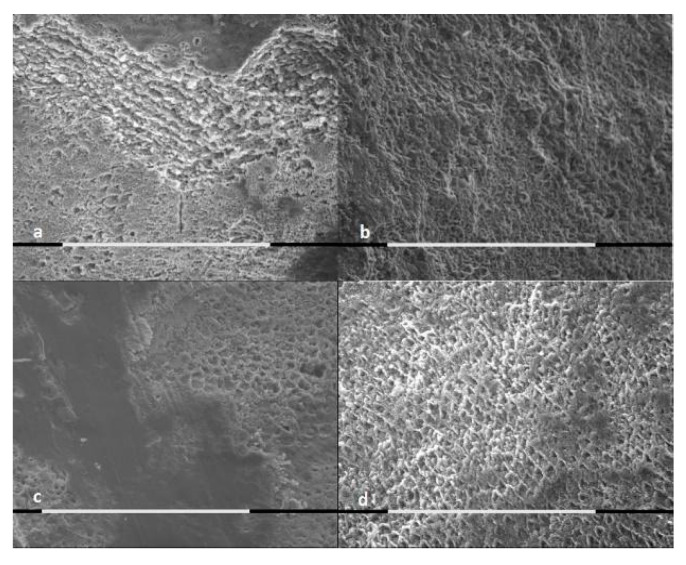
SEM pictures (800×, marker 100 μm) of enamel exposed to citric acid for 5 min: (**a**) enamel brushed with AZ ProExpert^®^ toothpaste; (**b**) untreated controls; (**c**) enamel treated with Colgate Total Original^®^ toothpaste; and (**d**) untreated controls.

**Figure 2:**
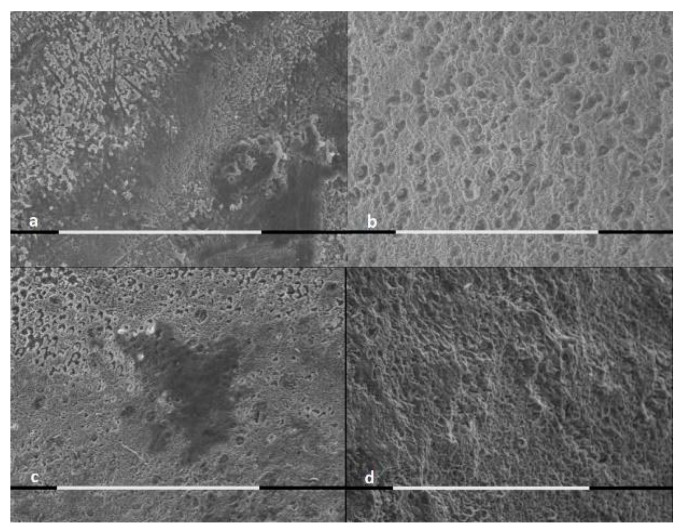
SEM pictures (800×, marker 100 μm) of enamel exposed to hydrochloric acid for 5 min: (**a**) enamel brushed with AZ ProExpert^®^ toothpaste; (**b**) untreated controls; (**c**) enamel treated with Colgate Total Original^®^ toothpaste; and (**d**) untreated controls.

**Figure 3:**
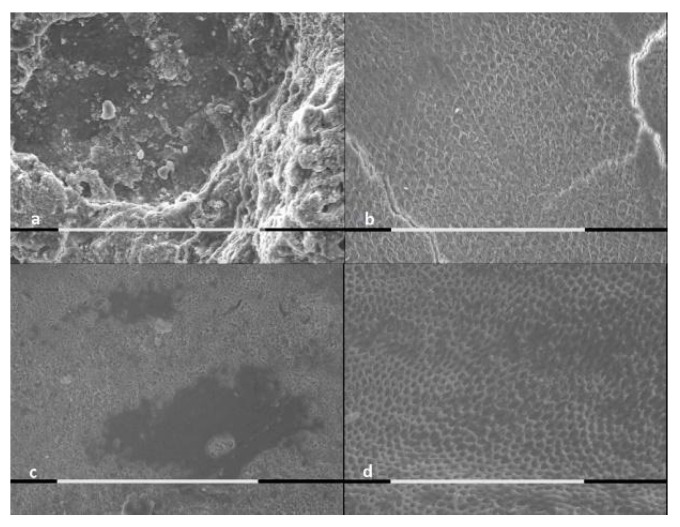
SEM pictures (800×, marker 100 μm) of enamel exposed to lactic acid for 5 min: (**a**) enamel brushed with AZ ProExpert^®^ toothpaste; (**b**) untreated controls; (**c**) enamel treated with Colgate Total Original^®^ toothpaste; and (**d**) untreated controls.

The SEM analysis of samples was performed for both treated and untreated cases, as shown by Figure 1, Figure 2 and Figure 3.

The control groups presented major extension of the demineralization areas, major surface roughness and major loss of mineral substance, in comparison to the samples treated with stannous or sodium fluoride.

The samples brushed with sodium fluoride showed areas free from demineralization, independently form the acid solution type.

The enamel brushed with stannous fluoride and exposed to lactic acid showed the presence of a layer of acid-resistant deposits.

According to the EDX microanalysis, calcium and phosphate ions were released by the samples treated with topical fluoride application. Their release could be due to superficial and sub-surface precipitates. Tin ion in the deposits of samples treated with stannous fluoride was not detected.

## 4. Discussion

Dental erosion has a multifactorial etiology: the main factors are a poor oral hygiene or an inadequate hygienic technique, a diet rich in carbohydrates or a frequent intake of soft drinks and some factors linked to systemic diseases. All these elements can expose the dental enamel to acid attacks [25,26,27] and increased dental permeability [28,29].

This study examined the citric acid, because of its presence in many drinks and wide use in food; the lactic acid, because of its involvement in the carious pathogenesis process; and the hydrochloric acid, because it is the most important component of gastric juices and it could be present in the oral cavity in conditions of vomiting or reflux [30,31,32,33].

Sodium fluoride-based toothpastes are the most widely used prevention tool for these acid exposure [17]. The present experimentation aimed to evaluate the effects of a single dose application of a new toothpaste with stannous fluoride on enamel exposed to acid solutions, in comparison with a sodium fluoride based one. Sodium fluoride and stannous fluoride act in a different way: the former operates more effectively as a result of the acid attack; the latter has an optimal action if applied before the erosive challenge. This means that the sodium fluoride has an optimal action during the first erosive attacks, while the stannous fluoride is more effective following repeated acid attacks, and therefore it is more suitable to inhibit enamel erosion in patients exposed to multiple erosive attacks [18]. The stannous fluoride is reported to offer protection against acid attacks due to the deposition of a barrier containing tin fluorophosphate, and its effect is active also at a 2.2 pH [21].

The spectrophotometric analyses performed in this study showed no statistically significant variations in the release of calcium and phosphate either between the two toothpastes or between the cases and the controls groups (*p* > 0.05). However, the null hypothesis could not be rejected.

A single dose application of a fluoride-based toothpaste could not prevent acid attack effects on enamel in in vitro conditions. However, the SEM morphological evaluation showed differences between the treated samples’ enamel surfaces and the controls’ samples enamel surfaces. The demineralized area extension, the surface roughness and the loss of mineral substance were reduced in samples treated with both fluoride-based toothpastes. The difference was that some specimens treated with the stannous fluoride-based toothpaste presented a deposited surface layer that was resistant to acid attack. All the samples treated with the sodium fluoride-based toothpaste, on the contrary, did not show a superficial deposit, even though they also had some areas protected by demineralization.

The absence of tin ion release in samples treated with stannous fluoride was detected with the EDX microanalysis. This can be attributed to the low concentration of the ion in the toothpaste and to the experimental protocol. The rinsing of the samples and the prolonged storing in artificial saliva, in fact, could be involved in the early release in solution.

### Limits of the Study

The samples storage in artificial saliva can simulate the oral environment only partially, though an in vivo study would be more reliable. Furthermore, the current investigation analyzed only the effects of a single brief acid attack simulation.

Following the results of the study and in particular the SEM/EDX analysis, it would be desirable to carry out further investigations about the protective effects of fluoride-based toothpastes, after multiple fluoride applications and repeated acid attack in oral cavity conditions.

## 5. Conclusions

A single dose of a fluoride-based toothpaste before different acids attack, in simulated oral cavity conditions, did not show a significant preventive effect in the present study, and the use of a daily fluoridated toothpaste alone may be ineffective in preventing enamel erosion. The application of stannous fluoride-based toothpaste created acid-resistant deposits that could prevent enamel demineralization.

## Figures and Tables

**Table 1:** Enamel samples in citric acid solution.

Toothpaste		Calcium (mg/dL)	Phosphate (mg/dL)
AZ ProExpert^®^	Cases (*n* = 6)	0.69 ± 0.32	0.88 ± 0.19
	Controls (*n* = 6)	0.46 ± 0.28	0.77 ± 0.29
Colgate Total Original^®^	Cases (*n* = 6)	0.70 ± 0.09	0.81 ± 0.15
	Controls(*n* = 6)	0.89 ± 0.07	0.77 ± 0.21

Calcium and phosphate release in AZ ProExpert^®^: toothpaste with stannous fluoride (SnF2); Colgate Total Original^®^: toothpaste with sodium fluoride (NaF) treated samples and controls.

**Table 2:** Enamel samples in hydrochloric acid solution.

Toothpaste		Calcium (mg/dL)	Phosphate (mg/dL)
AZ ProExpert^®^	Cases (*n* = 6)	0.81 ± 0.23	0.40 ± 0.16
	Controls (*n* = 6)	0.89 ± 0.21	0.37 ± 0.13
Colgate Total Original^®^	Cases (*n* = 6)	0.98 ± 0.09	0.59 ± 0.14
	Controls (*n* = 6)	0.91 ±0.12	0.48 ±0.078

Calcium and phosphate release in AZ ProExpert^®^: toothpaste with stannous fluoride (SnF2); Colgate Total Original^®^: toothpaste with sodium fluoride (NaF) treated samples and controls.

**Table 3:** Enamel samples in lactic acid solution.

Toothpaste		Calcium (mg/dL)	Phosphate (mg/dL)
AZ ProExpert^®^	Cases (*n* = 6)	0.73 ± 0.37	1.10 ± 0.99
	Controls (*n* = 6)	0.74 ± 0.21	0.45 ± 0.12
Colgate Total Original^®^	Cases (*n* = 6)	0.85 ± 0.26	0.41 ± 0.19
	Controls (*n* = 6)	0.62 ± 0.14	0.40 ± 0.11

Calcium and phosphate release in AZ ProExpert^®^: toothpaste with stannous fluoride (SnF2); Colgate Total Original^®^: toothpaste with sodium fluoride (NaF) treated samples and controls.
